# Factors influencing the implementation, adoption, use, sustainability and scalability of eLearning for family medicine specialty training: a systematic review protocol

**DOI:** 10.1186/s13643-016-0352-z

**Published:** 2016-10-19

**Authors:** Živa Cotič, Rebecca Rees, Petra A. Wark, Josip Car

**Affiliations:** 1 Global eHealth Unit, Department of Primary Care and Public Health, School of Public Health, Imperial College London, Charing Cross Campus, Reynolds Building, St Dunstan’s Road, London, W6 8RP UK; 2 Evidence for Policy and Practice Information and Co-ordinating Centre (EPPI-Centre), Social Science Research Unit, Department of Social Science, UCL Institute of Education, University College London, 18 Woburn Square, London, WC1H 0NR UK; 3 Lee Kong Chian School of Medicine, Imperial College and Nanyang Technological University, 3 Fusionopolis Link, #06-13, Nexus@One-North, South Tower, Singapore, 138543 Singapore

**Keywords:** eLearning, Postgraduate medical education, Family medicine, Qualitative systematic review

## Abstract

**Background:**

In 2013, there was a shortage of approximately 7.2 million health workers worldwide, which is larger among family physicians than among specialists. eLearning could provide a potential solution to some of these global workforce challenges. However, there is little evidence on factors facilitating or hindering implementation, adoption, use, scalability and sustainability of eLearning. This review aims to synthesise results from qualitative and mixed methods studies to provide insight on factors influencing implementation of eLearning for family medicine specialty education and training. Additionally, this review aims to identify the actions needed to increase effectiveness of eLearning and identify the strategies required to improve eLearning implementation, adoption, use, sustainability and scalability for family medicine speciality education and training.

**Methods:**

A systematic search will be conducted across a range of databases for qualitative studies focusing on experiences, barriers, facilitators, and other factors related to the implementation, adoption, use, sustainability and scalability of eLearning for family medicine specialty education and training. Studies will be synthesised by using the framework analysis approach.

**Discussion:**

This study will contribute to the evaluation of eLearning implementation, adoption, use, sustainability and scalability for family medicine specialty training and education and the development of eLearning guidelines for postgraduate medical education.

**Systematic review registration:**

PROSPERO http://www.crd.york.ac.uk/PROSPERO/display_record.asp?ID=CRD42016036449

**Electronic supplementary material:**

The online version of this article (doi:10.1186/s13643-016-0352-z) contains supplementary material, which is available to authorized users.

## Background

Developing an efficient and sufficient global health workforce is one of the most pressing global health issues. In 2013, the World Health Organization (WHO) and the Global Health Workforce Alliance reported a shortage of 7.2 million health workforce which is expected to increase to 12.9 million by 2035 [1]. This shortage of health workers is additionally aggravated by inefficient delivery of health education. The content, organisation and delivery of health education often fail to equip health workers with the skills, competencies, experience and expectations needed to satisfy the changing population health needs [2].

Specific sets of specialities have proven to be most problematic in terms of developing and retaining workforce, namely family medicine specialists or general practitioners. In practically every country, the balance between family medicine specialists and other specialists is disproportionate, since the number of specialists is increasing faster than the number of generalists. This is mostly due to declining interest in family medicine, the amount of work and the remuneration gap between family medicine specialist and other specialists [3].

To address these issues, eLearning (electronic learning) has increasingly been used in health professional education. eLearning can be defined as ‘an approach to teaching and learning, representing all or part of the educational model applied, that is based on the use of electronic media and devices as tools for improving access to training, communication and interaction and that facilitates the adoption of new ways of understanding and developing learning’ [4].

Advancements in eLearning technologies have produced various forms of eLearning modalities such as computer-based simulations, virtual patients and internet-based learning [5]. The development of these technologies could promote global knowledge sharing and contribute to the training of health care professionals in places with acute manpower shortage and in resource-constrained settings [6, 7] as well as in high-income countries [2]. Other benefits to the use of eLearning technologies include a potential reduction in the cost related to course delivery, the capacity to transfer knowledge without any space and time constraint and the ability to personalise course contents to suit learners’ needs [1].

Past reviews on eLearning have mainly focused on the impact and effectiveness of internet-based learning in undergraduate education [5, 8] and postgraduate education [9]. In 2014, two systematic reviews looking at online and offline learning for undergraduate health professional education have concluded that eLearning could be as effective as traditional learning (i.e. classroom-based face-to-face learning) [6, 7]. Simulation for undergraduate and graduate medical simulation has been shown to be effective for developing psychomotor and communication skills [10]. In the context of continuing medical education, internet-based programmes have been shown to be as effective for knowledge acquisition as traditional programmes [9].

While a number of qualitative studies had been conducted on the topic of eLearning [11–14], including a qualitative review limited to the UK [15], the evidence base for eLearning is still largely underdeveloped.

Systematic review methods can be applied to qualitative study findings to gain insight into people’s experiences and perspectives and to better understand the nature of material and socio-cultural influences on intervention effectiveness, as well as better understand causal pathways [16] or delineate a more complete picture of the phenomenon in question [17]. Qualitative studies take an interpretive approach. They often collect data with flexible methods such as open-ended interviewing and/or observations and apply qualitative analysis techniques to provide insights into important concepts and to develop theories. The synthesis of such studies tends also to work mainly with qualitative data (from study reports) and configure study findings to produce themes which may be ordered to describe variation within a phenomenon or developed into new theory [18].

An initial scoping of the literature has identified eight published studies on barriers and/or facilitators to eLearning for family medicine specialty training and education amounting to 343 participants. All of the studies focused on eLearning requiring internet connectivity (online eLearning). However, they explored different modalities of online eLearning: virtual communities of practice [19, 20], e-conferencing [21], immersive virtual (3-D) environment [22], simulation [23] and subject specific online modules [24–26]. Factors (barriers and facilitators) were explored using either quantitative methodology [19, 21, 24] or mixed methods [20, 22, 23, 25, 26]. While several systematic reviews are currently underway to investigate the effectiveness of eLearning, no review yet has been conducted to systematically evaluate the factors influencing the implementation, adoption, use, sustainability and scalability of eLearning for family medicine specialty training. A related review is also underway to examine the processes involved in the delivery of mLearning in health professional training.

## Methods/design

### Review aims and research questions

This proposed systematic review aims to draw implications from empirical research exploring the processes involved in the delivery of eLearning in the field of family medicine specialty training, or has sought the perspectives of learners, educators and others with experience of eLearning in this field. Themes arising from a synthesis of the findings of this research will be used to consider:

I. The eLearning pedagogies that teachers might adopt to optimise knowledge formation and knowledge retention in family medicine trainees
II. Strategies that might ameliorate negative and enhance positive factors potentially influencing eLearning for health professional education

The broad research question for this review is:

I. What are the views of educators, learners and other key actors with experience in eLearning for family medicine specialty training about perceived factors that facilitate or hinder its implementation, adoption, scalability, sustainability and educational impact?

### Research framework

To better understand eLearning, it is important to explore it in terms of the underlying assumptions about art or science of teaching and educational methods (pedagogy) [27]. We have chosen Laurillard’s Conversational Framework (LCF) as conceptual framework to be used in this review. LCF describes interactions between learners, peers and teachers in formal and informal learning contexts (Laurillard 2007). It provides a detailed description of components affecting the motivation of the learners in collaborative learning environments, such as specialty training setting, using a combination of social learning theories, constructionism and instructionism. This framework sees all participants in the learning process as being influenced by each other’s’ presentations of concepts and responses to learning tasks and goals. It also attempts to capture ideas about the influence learning environment designs as well as learning environment practice.

This framework, however, does not consider the influence that socio-cultural and policy-related constructs can have on the eLearning process in the context of postgraduate medical education and specialty training. It has been advocated that medical education researchers and curriculum developers consider the external and internal, implementation, relevant experience and impact factors when designing technology-based interventions [28].

Therefore, in line with the requirement that medical education researchers and curriculum developers consider implementation and relevant experience and not only the impact factors [28], theory of implementation needs to be considered. Theory of implementation uses concepts and arguments to predict or explain how courses of action taken to put an idea, decision, procedure or programme into use result in observed patterns of initial use or early use. Theory of implementation is threefold; it consists of top-down, bottom-up and combined or synthesised approaches [29, 30].

In his paper on policy implementation, Najam sees implementation as ‘a dynamic process of negotiation between multiple actors, operating at multiple levels, within and between multiple organizations’ [31]. To make sense of complex implementation processes, Najam developed the 5C Protocol. The protocol’s ‘5Cs’ stand for content, context, commitment, capacity and clients and coalitions. All five variables are linked to each other. Najam advises that rather than looking at the variables themselves, researchers should be interested in cataloguing the strengths and influences of the variables on an implementation endeavour and identifying connections between them on the basis of their potential to improve the effectiveness of the endeavour. The role of implementation analysis is prescriptive and can lead to policy change [31].

Using implementation theory and Najam’s 5C protocol might help systematically explore the roles and views of all stakeholders involved in eLearning implementation in the context of family medicine specialty training and education.

### Criteria for considering studies for this review

Given that the extent of the evidence base that addresses the above research question is unknown, this review will have two stages: a systematic ‘mapping’ of research evidence, followed by an in-depth analysis and synthesis. The criteria below are for the systematic map, unless otherwise specified. The synthesis will address the following themes, contexts and factors, with distinct analyses of subsets of the full evidence-base to give appropriate attention to the phenomena of interest.

#### Types of studies

We will include studies that:

I. Examine peoples’ perspectives on and experiences of eLearning (see ‘Types of intervention’ below) so as to produce findings about perceived factors which facilitate/enhance or hinder its implementation, adoption, scalability, sustainability and educational impact.
  ○ For in-depth review and synthesis, depending on the quantity and nature of the research found in the systematic map, it may be helpful to restrict studies to those that meet the inclusion criteria above and below but also collect and analyse data primarily through the use of qualitative methods. This would include, but not be limited to, studies underpinned by theoretical frameworks such as phenomenology, ethnography and grounded theory, action and narrative research and case studies. Qualitative methods for data collection would include focus groups, in-depth individual interviews and observations.
II. Have an abstract published in English, Italian, Slovene or any other language spoken by the review team.

We will exclude:

I. Systematic reviews, however, their reference lists will be screened for suitable studies.
II. Commentaries, letters, editorials and other kinds of literature reviews.

#### Types of participants

We will include studies with participants who are or have been enrolled in family medicine specialty training in any geographical setting (e.g. low- and middle- and high-income countries) and any educational setting (e.g. university, laboratory, medical ward, community).

#### Types of intervention

We will include studies that explore eLearning in family medicine specialty training and education. eLearning is defined as an ‘approach to teaching and learning, representing all or part of the educational model applied, that is based on the use of electronic media and devices as tools for improving access to training, communication and interaction and that facilitates the adoption of new ways of understanding and developing learning’ [4], which includes a range of modalities such as mLearning, computer-based simulations, virtual patients and internet-based learning.

### Search methods for identification of studies

#### Electronic searches

The searches for this review have been run as one component of a larger series of evidence synthesis reviews on eLearning for health professional education conducted in collaboration with the World Health Organization for which a common search strategy has been developed.

Searches that cover all of the above topics (eLearning for health professional education) have been run on the following bibliographic databases:

- Systematic review registers
  - ○ The Cochrane Library (Cochrane Database of Systematic Reviews, Cochrane Central Register of Controlled Trials (CENTRAL), Cochrane Methodology Register)
- Education focused databases
  - ○ Education Resources Information Centre (ERIC)
- Health focused databases
  - ○ Cumulative Index to Nursing and Allied Health Literature (CINAHL)
  - ○ EMBASE (Elsevier)
  - ○ MEDLINE (Ovid)
- Other databases
  - ○ PsycINFO
  - ○ Web of Science Core Collection (Thomson Reuters)

When searching these databases, sets of terms have been identified from each database’s controlled classifying terminology for each of the main concepts found within all of the reviews research questions (eLearning, health professionals, postgraduate education). These sets of terms have then been combined to find only those records that have been classified with a term relating to all of the concepts. Searches using free-text terms have also been ran to help identify relevant studies that, for whatever reason, have not been allocated controlled terms. Searches have been limited to items published from 1990 to 5 March 2015 (see Additional file 1).

#### Searching other sources

The references of included studies will be screened, and unpublished and other studies that might be relevant will also be sought from the authors of included studies and from others active in this area of research.

### Data collection and analysis

All records of studies that are identified by these above specified searches will be uploaded to EPPI-Reviewer 4, specialist systematic review software [32]. The studies will be deduplicated. Separate searches for the purposes of this review will then be conducted within EPPI-Reviewer to identify relevant qualitative studies. Further sets of terms will be combined, using the software’s search function, which looks for each search term within a record’s title and/or its abstract. Sets of terms will be developed to cover the concepts central to this review (eLearning, perspectives, experiences) and combined to identify a set of records to screen.

#### Selection of studies

The search results will be screened to identify studies to include in an initial systematic map. These studies will be coded (see below) to provide an overview of the nature and extent of the literature that addresses the review’s research questions. Following consideration of the range of study designs seen in the map, the mapped studies may be screened again using a refined set of inclusion criteria so as to identify studies that have applied certain aspects of study design, with only these studies then being described further, being fully appraised for their methodological quality and having their findings extracted for inclusion in a synthesis.

All review authors will initially work together with a sample set of identified studies. These will be used to pilot the inclusion criteria and then to reach a high level of agreement between all the review authors in using the criteria to determine a study’s eligibility for inclusion. The titles and abstracts of each report retrieved from the search strategy and the additional sources described will then be screened independently by two review authors, who will discuss all cases where they initially disagree on whether or not a report should be included. A third review author will help decide upon inclusion of a report in all cases where the two initial reviewers cannot agree. After initial screening, full texts of studies will be obtained and then screened by two review authors working independently.

#### Data extraction and management

All studies that are included in the systematic map will be described according to a standardised coding system that will be modified corresponding to the purposes of this review [33].

Codes applied to capture the key characteristics of relevant studies are likely to include but will not be limited to:

- Codes to describe the study context and population, including
  - ○ The country setting (e.g. low- and middle-income countries, high-income countries)
  - ○ The educational setting (e.g. specialty training and education)
  - ○ Relevant defining features of the sampled population (e.g. gender, age, years of education/training, type/level of training, years of experience)
- Codes to describe the intervention under study, including
  - ○ Study aims/research questions
  - ○ The eLearning modality (e.g. simulation, internet-based learning, virtual reality)
  - ○ Learning platform (e.g. chat group, e-book, web-based module, mobile application)
  - ○ Component of intervention (e.g. skills training, cognitive/knowledge based training, provision of resources or supplementary information, services rendered)
  - ○ Duration of intervention (e.g. <1 month, 1–6 months, 7–12 months, >12 months)
- Codes to describe the study design, including:
  - ○ The type of data collection method used (e.g. survey with open-ended questions; observational study using case study techniques; in-depth individual interviews; focus groups)
  - ○ Sampling approach (e.g. convenience sampling, random sampling, purposive sampling, snowball sampling, theoretical sampling)
  - ○ The sampling frame (e.g. course enrolment list, directory of family medicine specialty trainees working in the hospital)
  - ○ The sample size (e.g. <10, 11–20, 21–50, 51–100, >100)
  - ○ The type of analysis (qualitative only, qualitative and quantitative)
  - ○ Type of outcomes (e.g. attitudes, skills, knowledge, experiences, feelings)

All studies that are included in the in-depth review will be described further using additional, standard questions, such as those used in previous reviews of intervention processes and stakeholder perspectives [33, 34].

#### Quality and certainty in review findings appraisal

The quality of studies included in the in-depth review will be examined using a modified version of the Critical Appraisal Skills Program (CASP) quality assessment tool for qualitative studies [35]. Studies that meet the inclusion criteria will be included in the review regardless of the study quality, and quality assessment findings will be presented along with other descriptive characteristics for each included study. Using an overall assessment of the methodological strengths and limitations of the studies, the authors will make an overall judgement of the papers in the review. The Confidence in the evidence from reviews of Qualitative research approach (CERQual) will be followed and used to guide assessments of the certainty of the findings from the review’s synthesis [36].

#### Data synthesis

The findings and contextual detail from each included study will be entered into a framework synthesis. This involves the construction of thematic categories from the findings of included studies through the use of a matrix within which the findings are coded [37]. A distinctive feature of framework synthesis is that it utilises an a priori ‘framework’ as a starting point for the synthesis. An initial, ‘good enough’, framework is developed by a review team’s reading and discussion of theoretical material that relates to the concepts in the review’s research question. The synthesis approach is then deductive [38], with reviewers attempting to match the findings of included studies with the different aspects of their initial conceptual frame. When the findings are found to address an area not covered in the initial frame, the frame is modified, until the frame addresses all of the themes arising from the included studies. Additional work uses other dimensions of the included studies to ensure the synthesis takes into account the variation across and within different study populations and contexts. This review will apply the following five stages of framework analysis [39]:

1. Familiarisation—this stage involves the authors being immersed in the data by reading and studying the papers retrieved with the aims and objectives of the review and listing key ideas and recurrent themes.
2. Identifying a thematic framework—this process involves identification of key issues, concepts and themes using the a priori issues and questions raised from the aims and objectives of the review and experiences and perspectives that recur in the data. This stage results in the formulation of a detailed index of the data, in which data is labelled in manageable chunks for subsequent retrieval and exploration. At this stage, we may incorporate into a framework, aspects of Laurillard’s framework [27] so as to identify the different pedagogic forms of eLearning that have previously been described by teachers as optimising learning and aspects of Najam’s 5C protocol [31] to allow consideration of a wider range of eLearning stakeholder experience.
3. Indexing—during this stage, the thematic framework is applied by annotating transcripts of findings from included studies with codes from the index and supporting them with short text descriptors to substantiate the index heading. At least two review authors will independently read and re-read the selected studies and apply the review’s initial framework. The framework can be applied by moving between the data and the themes covered by the framework and searching for additional themes until all of the studies have been reviewed, in an iterative manner. At this stage, the definitions and boundaries of each of the emerging themes will be discussed among all review authors and revision of the model will be conducted in line with the ideas and categories that emerge from this process.
4. Charting—the data is then re-arranged according to the relevant part of the thematic framework, and the information is distilled and summarised in the form of charts. At this stage, it is likely that a chart is created for each key subject area or themes from several respondents or papers through abstraction and synthesis of the data. The charts will contain distilled summaries of evidence from different perspectives and involve a high level of abstraction and synthesis.
5. Mapping and interpretation—the charts will be used together with the research objectives and themes that have emerged, to define concepts and explain the findings through clarification of the phenomena, creation of typologies and finding associations between themes.

## Discussion

An understanding of the factors (barriers and facilitators) influencing eLearning implementation, adoption, scalability, sustainability and educational attainment is necessary for further development and improvement of implementation strategies. The findings of this review will contribute to the planning and design of effective eLearning for family medicine specialty training and education and the development of eLearning guidelines. In addition, we will identify gaps in literature to inform future research and policy development for wider implementation of eLearning.

## Presenting and reporting the results

This protocol will adhere to the Preferred Reporting Items for Systematic Review and Meta-Analysis Protocols 2015 Statement (PRISMA-P) [40] (Additional file 2). The results of the review will be presented according to the Preferred Reporting Items for Systematic Reviews and Meta-Analyses (PRISMA) guidelines [41]. We will produce a complete PRISMA flow chart and include a table of all included studies in the final review.

## Additional files

Additional file 1: Medline (Ovid). (DOCX 16 kb)
Additional file 2: PRISMA-P (Preferred Reporting Items for Systematic review and Meta-Analysis Protocols) 2015 checklist: recommended items to address in a systematic review protocol*. (DOC 82 kb)

## Abbreviations

CASP: Critical Appraisal Skills Program
CERQual: Confidence in the evidence from reviews of Qualitative research approach
eLearning: Electronic learning
LCF: Laurillard’s Conversational Framework
WHO: World Health Organization
