# Supplementary material for: Factors influencing the implementation, adoption, use, sustainability and scalability of eLearning for family medicine specialty training: a systematic review protocol
Source: Syst Rev. 2016 Oct 19;5:179. doi: 10.1186/s13643-016-0352-z (PMC5070227; doi:10.1186/s13643-016-0352-z)
Supplement: Additional file 1: — Medline (Ovid). (DOCX 16 kb) [file 13643_2016_352_MOESM1_ESM.docx]

. Medline (Ovid)

Date: 2015-02-27

Number of hits: 44,561

| 1. exp education, professional/ not education, veterinary/  2. Education, Predental/  3. Education, Premedical/  4. exp Students, Health Occupations/  5. ((medic* or premedic* or dent* or laborator* or predent* or midwi?e* or nurs* or nutrition* or orthop* or podiat* or pharmac* or psycholog* or psychiatr* or health or healthcare or occupational therap* or physiotherap* or physical therap* or clinical or surg* or radiolog* or obstetric* or gyn?ecolog* or orthodont* or An?esthesi* or Dermatolog* or Oncolog* or Rheumatolog* or Neurolog* or Patholog* or P?ediatric* or Cardiolog* or Urolog*) adj3 (student* or graduate* or undergraduate* or staff or personnel or practitioner* or clerk* or fellow* or internship* or residen* or educat* or train* or novice* or tutor*)).tw,kf.  6. or/1-5  7. Computer-Assisted Instruction/  8. exp Internet/  9. Computer Simulation/  10. Patient Simulation/  11. software/  12. Mobile Applications/  13. User-Computer Interface/  14. Video Games/  15. Web Browser/  16. Education, Distance/  17. Computers/  18. exp Microcomputers/  19. exp Cell Phones/  20. Games, Experimental/  21. exp Models, Anatomic/  22. Audiovisual Aids/  23. Educational Technology/  24. Electronic Mail/  25. exp Telemedicine/  26. Telenursing/  27. Telecommunications/  28. Webcasts/  29. exp Videoconferencing/  30. ((computer* or digital* or hybrid or blended or mixed mode or distance or remote* or electronic or mobile or online* or interactiv* or multimedia or internet or web* or virtual* or game* or gaming or Videogame* or Videogaming) adj3 (classroom* or course* or educat* or instruct* or learn* or lecture* or simulat* or train* or teach* or tutor* or platform*)).tw,kf.  31. (Simulat* adj3 (course* or educat* or instruct* or learn* or train* or teach* or platform* or high-fidelity)).tw,kf.  32. e-learn*.tw,kf.  33. elearn*.tw,kf.  34. m-learn*.tw,kf.  35. mlearn*.tw,kf.  36. smartphone*.tw,kf.  37. smart-phone*.tw,kf.  38. ((mobile or cell) adj2 phone*).tw,kf.  39. iphone*.tw,kf.  40. android*.tw,kf.  41. ipad*.tw,kf.  42. Personal digital assistant*.tw,kf.  43. handheld computer*.tw,kf.  44. Mobile App?.tw,kf.  45. Mobile Application?.tw,kf.  46. webcast*.tw,kf.  47. webinar*.tw,kf.  48. flipped classroom*.tw,kf.  49. Serious game*.tw,kf.  50. Serious gaming.tw,kf.  51. Patient Simulat*.tw,kf.  52. Virtual patient*.tw,kf.  53. ((educat* or instruct* or learn* or simulat* or train* or teach* or interactiv*) adj2 technolog*).tw,kf.  54. Massive Open Online Course?.tw,kf.  55. Mooc?.tw,kf.  56. (Canvas network or Coursera or Coursesites or edx or Futurelearn or iversity or miriada x or moodle or novoed or openlearning or open2study or plato or spoc or udacity or pingpong).tw,kf.  57. or/7-56  58. 6 and 57  59. Education.fs.  60. Education/  61. Teaching/  62. Learning/  63. exp Inservice Training/  64. Curriculum/  65. educat*.tw,kf.  66. learn*.tw,kf.  67. train*.tw,kf.  68. instruct*.tw,kf.  69. teach*.tw,kf.  70. or/59-69  71. Health Personnel/  72. exp Allied Health Personnel/  73. Anatomists/  74. "Coroners and Medical Examiners"/  75. exp Dental Staff/  76. exp Dentists/  77. Health Educators/  78. Infection Control Practitioners/  79. Medical Laboratory Personnel/  80. exp Medical Staff/  81. exp Nurses/  82. exp Nursing Staff/  83. Personnel, Hospital/  84. Pharmacists/  85. exp Physicians/  86. Physician*.tw,kf.  87. Doctor*.tw,kf.  88. Nurs*.tw,kf.  89. Surg*.tw,kf.  90. Health Personnel.tw,kf.  91. healthcare professional*.tw,kf.  92. radiolog*.tw,kf.  93. dentist*.tw,kf.  94. Pharmacist*.tw,kf.  95. Hospital Administrator*.tw,kf.  96. Podiatr*.tw,kf.  97. Psycholog*.tw,kf.  98. Psychiatr*.tw,kf.  99. An?esthesi*.tw,kf.  100. Clinician*.tw,kf.  101. Dermatolog*.tw,kf.  102. General practioner*.tw,kf.  103. Cardiolog*.tw,kf.  104. Oncolog*.tw,kf.  105. Rheumatolog*.tw,kf.  106. Neurolog*.tw,kf.  107. Patholog*.tw,kf.  108. P?ediatric*.tw,kf.  109. Physiotherap*.tw,kf.  110. Physical therap*.tw,kf.  111. Occupational therap*.tw,kf.  112. dieti?ian*.tw,kf.  113. Dietetic*.tw,kf.  114. midwi?e*.tw,kf.  115. nutrition*.tw,kf.  116. orthopti*.tw,kf.  117. obstetric*.tw,kf.  118. gyn?ecolog*.tw,kf.  119. orthodont*.tw,kf.  120. Urolog*.tw,kf.  121. or/71-120  122. Health Occupations/  123. exp Allied Health Occupations/  124. Biomedical Engineering/  125. Chiropractic/  126. exp Dentistry/  127. exp Evidence-Based Practice/  128. exp Medicine/  129. exp Nursing/  130. Dietetics/  131. Optometry/  132. Orthoptics/  133. exp Pharmacology/  134. exp Pharmacy/  135. Podiatry/  136. Psychology, Medical/  137. Serology/  138. Specialization/  139. exp Surgical Procedures, Operative/  140. exp Radiography/  141. or/122-140  142. 121 or 141  143. 57 and 70 and 142  144. Psychomotor Performance/  145. motor skills/  146. ((psychomotor or procedural or technical) adj3 skill*).tw,kf.  147. (psychomotor adj3 performance).tw,kf.  148. or/144-147  149. 6 and 148  150. 58 or 143 or 149  151. limit 150 to yr="1990 -Current" |
| --- |
